# A mouse model of gestational diabetes shows dysregulated lipid metabolism post-weaning, after return to euglycaemia

**DOI:** 10.1038/s41387-022-00185-4

**Published:** 2022-02-15

**Authors:** Samuel Furse, Denise S. Fernandez-Twinn, Jessica H. Beeson, Davide Chiarugi, Susan E. Ozanne, Albert Koulman

**Affiliations:** 1 Core Metabolomics and Lipidomics Laboratory, Wellcome-MRC Institute of Metabolic Science, University of Cambridge, Addenbrooke’s Treatment Centre, Keith Day Road Cambridge, CB2 0QQ Cambridge, UK; 2 Wellcome-MRC Institute of Metabolic Science and Medical Research Council Metabolic Diseases Unit, University of Cambridge, Cambridge, UK; 3 Biological chemistry group, Jodrell laboratory, Royal Botanic Gardens Kew, Cambridge, UK; 4 Wellcome-MRC Institute of Metabolic Science, University of Cambridge, Addenbrooke’s Treatment Centre, Keith Day Road Cambridge, CB2 0QQ Cambridge, UK; 5 Bioinformatics and Biostatistics Core, Wellcome-MRC Institute of Metabolic Science and Medical Research Council Metabolic Diseases Unit, Addenbrooke’s Treatment Centre, Keith Day Road Cambridge, CB2 0QQ Cambridge, UK

**Keywords:** Lipidomics, Gestational diabetes

## Abstract

**Background:**

Gestational diabetes is associated with increased risk of type 2 diabetes mellitus and cardiovascular disease for the mother in the decade after delivery. However, the molecular mechanisms that drive these effects are unknown. Recent studies in humans have shown that lipid metabolism is dysregulated before diagnosis of and during gestational diabetes and we have shown previously that lipid metabolism is also altered in obese female mice before, during and after pregnancy. These observations led us to the hypothesis that this persistent dysregulation reflects an altered control of lipid distribution throughout the organism.

**Methods:**

We tested this in post-weaning (PW) dams using our established mouse model of obese GDM (high fat, high sugar, obesogenic diet) and an updated purpose-built computational tool for plotting the distribution of lipid variables throughout the maternal system (Lipid Traffic Analysis v2.3).

**Results:**

This network analysis showed that unlike hyperglycaemia, lipid distribution and traffic do not return to normal after pregnancy in obese mouse dams. A greater range of phosphatidylcholines was found throughout the lean compared to obese post-weaning dams. A range of triglycerides that were found in the hearts of lean post-weaning dams were only found in the livers of obese post-weaning dams and the abundance of odd-chain FA-containing lipids differed locally in the two groups. We have therefore shown that the control of lipid distribution changed for several metabolic pathways, with evidence for changes to the regulation of phospholipid biosynthesis and FA distribution, in a number of tissues.

**Conclusions:**

We conclude that the control of lipid metabolism is altered following an obese pregnancy. These results support the hypothesis that obese dams that developed GDM maintain dysregulated lipid metabolism after pregnancy even when glycaemia returned to normal, and that these alterations could contribute to the increased risk of later type 2 diabetes and cardiovascular disease.

## Introduction

Gestational diabetes mellitus (GDM) is the most common complication of pregnancy and is known to be associated with an increased risk of metabolic disease *post partum*. Obesity is a major risk factor for GDM, with around a third of women with a BMI > 30 developing GDM during their first pregnancy [1–3] compared to around 8% in the general UK population [4]. Women who develop gestational diabetes are at increased risk of type 2 diabetes mellitus (T2DM) in the decade after delivery [5–8]. Furthermore, a systematic review of over 5 million women in 9 studies found that women who previously had GDM had a two-fold increased risk of cardiovascular events independent of a progression to T2DM [9].

Studies of lipid metabolism during pregnancy and before the diagnosis of GDM in humans have shown that several lipid pathways are dysregulated before the hyperglycaemia associated with GDM becomes established [10–12]. Specifically, serum-serum comparisons of control and GDM groups have identified differences in triglyceride (TG), phosphatidylcholine (PC) and sphingomyelin (SM) lipid classes [11–13]. These include lipids involved in energy storage and distribution (TGs) as well as membrane structure (PC, SM). These patterns were observed in both a healthy BMI [12] and an obese [11] human cohort. Most of the variables identified had several olefin bonds, indicating that the supply and distribution of polyunsaturated fatty acids may be important. As this dysregulation of lipid metabolism in humans occurs 10 weeks before diagnosis of gestational diabetes and includes alterations in the metabolism of both structural lipids and triglycerides, the evidence suggests there are systemic dysregulations in lipid metabolism that could contribute to the development of GDM. Using a mouse model of obese GDM, we recently showed that, similar to humans, lipid metabolism as measured in serum, was dysregulated even before pregnancy [14]. Furthermore, we also demonstrated major changes in lipid classes with advancing pregnancy [14], plausibly an effect of hyperglycaemia and impaired glucose tolerance [15] over and above that which occurs in a lean pregnancy [16].

**Fig. 1:**
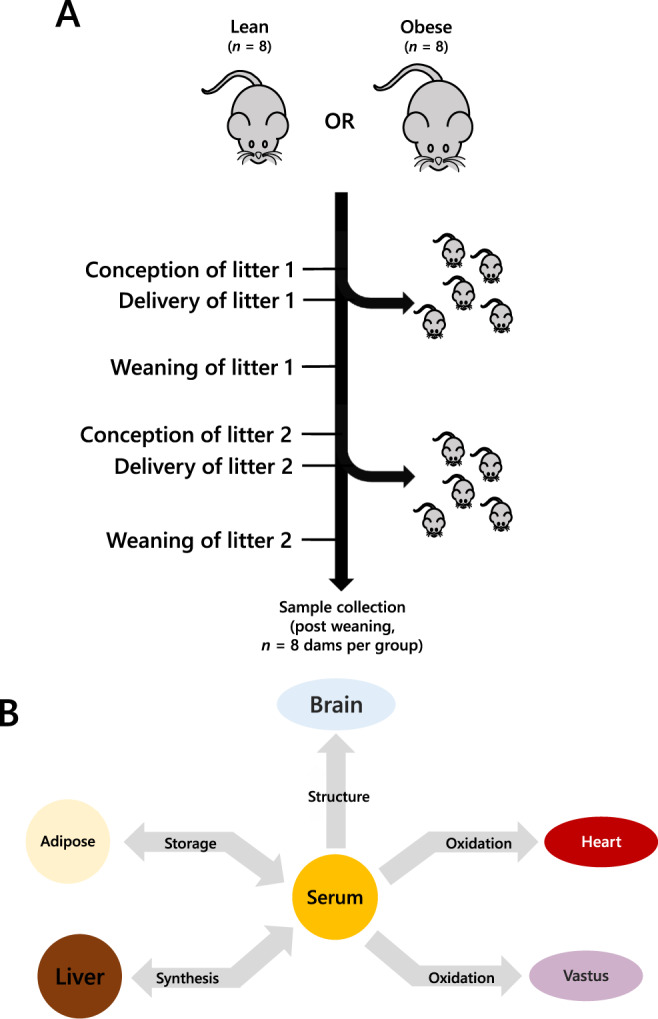
The tissue network of the mouse model of obese-GDM used in the present study. **A** Schematic representation of the mouse model showing the groups, pregnancies and weaning. **B** The network that describes the lipid traffic associated with this model, including tissues whose activity is typically associated with diet-driven diabetes. The termini represent traffic flow from synthesis (liver), for structural purposes (CNS), fatty acid oxidation (heart, vastus, liver) and storage (adipose). This metabolic relationship between tissues was used as the structure of the network for all analyses in the present study. Lean refers to mice fed exclusively a diet of normal chow *ad libitum* whereas Obese refers to mice fed a high fat diet drawn from mainly dairy fat sources.

Studies in humans have shown that maternal obesity not only increases the mothers’ risk of GDM [3, 4] but also drives the programming of cardio-metabolic disease in offspring [17, 18]. Animal studies clearly support this programmed risk to the offspring [19, 20]. Women with a history of gestational diabetes mellitus have a 2-fold higher CVD risk than the background population [21] highlighting a major health problem. Cardiovascular disease is a major killer of women in the UK and the USA with nearly double the rate of mortality as breast cancer [22]. Human studies have shown that an altered lipid metabolism in women increases their risk of cardiovascular disease [23] and is associated with prior exposure to GDM [24]. In the current study we test the hypothesis that GDM is accompanied by changes in lipid metabolism that remain after pregnancy, when the hyperglycaemia associated with GDM has disappeared. To test this, we used an established mouse model of maternal high-fat-diet-induced obesity in which dams display impaired glucose tolerance during pregnancy, that, as is typical with GDM, resolves after delivery [15, 25, 26] (Fig. 1A). We profiled glycaemia and insulinaemia in the post-weaning mice and collected a range of metabolically active tissues to determine organismal lipid metabolism after weaning (22 days *post partum*, Fig. 1B). We capitalised on an updated version of Lipid Traffic Analysis [27, 28] (LTA, v2.3) to analyse lipidomics data from the tissues to do a system-level characterisation of the number, type and abundance of lipid variables in and between compartments in the two groups of female mice. LTA is a powerful tool to test our hypothesis because it uses the spatial distribution of lipids to test how the control of lipid metabolism differs between the two groups.

## Materials and methods

### Animal model

All procedures were conducted in accordance with the UK Home Office Animal (Scientific Procedures) Act 1986 and following local ethics committee approval at the University of Cambridge. Animals were maintained at the University’s biomedical research facility as described previously [15, 25, 26]. Briefly, female C57BL/6 J mice were fed either a control (RM1) or an obesogenic high-fat-high-sugar diet from weaning and throughout 2 pregnancy and lactation cycles (Fig. 1A, both diets manufactured by Special Dietary Services Ltd; Witham, UK). As described previously [15, 29], the composition of the respective diets are as follows: Control diet [~7% simple sugars, 3% fat, 50% polysaccharide, and 15% protein (*w/w*)]; Obesogenic diet [high-fat diet: ~10% simple sugars, 20% animal lard, 28% polysaccharide, and 23% protein (*w/w*) supplemented with sweetened condensed milk supplied in a glass jar (~55% simple sugar, 8% fat, and 8% protein (*w/w*)) and micronutrient mineral mix]. Proven breeders were rested for 1-3 weeks between weaning of the first litter and before mating for the second (experimental) pregnancy. The timing of this was determined when the control dams had achieved a total body-fat mass of no more than 5 g, and the obese exceeded 10 g of total fat mass as assessed by time domain nuclear magnetic resonance (TD-NMR) (Mini-spec TD-NMR, Bruker UK Ltd). The sires used were all C57BL/6 J mice of 12-24 weeks of age. To negate the effects of confounding paternal nutritional programming [27, 30, 31] the sires were only fed the control diet apart for the duration of mating. At conception, as indicated by a vaginal plug, the dams were singly housed throughout pregnancy. Immediately after weaning, the dams remained singly housed for 48 hours to measure food intake by weighing the contents of the diet hopper (and the pot of condensed milk) at the start and end of the 48-hour period. Pellet (and milk) intake was then averaged over the period to determine daily food intake. *n* = 8 dams per group were used. Just before killing, body composition was measured by Time Domain Nuclear Magnetic Resonance (TD-NMR) (Mini-spec TD-NMR, Bruker UK Ltd). Dams were culled by a rising concentration of carbon dioxide followed by cardiac puncture for blood collection and tissue dissection. Brain, adipose tissue, liver, heart and vastus lateralis muscle were weighed and flash frozen in liquid nitrogen before being stored at −80 °C until lipid analysis.

### Glucose tolerance test

On the day of weaning (21 days *post partum*), dams were fasted for 16 h, beginning at 16:30. Dams were placed individually into a clean cage with access to water. Blood was drawn from the tail for basal (0 min) glucose measurements (AlphaTRAK2, Zoetis, USA). Dams were then injected intraperitoneally (i.p.) with 1 g/kg glucose and further tail blood glucose measurements were made at timed intervals after injection (15 min, 30 min, 45 min, 60 min, 90 min and 120 min). Blood was also collected at 0 min, 15 min and 30 min time points into haematocrit sodium-heparin capillary tubes (Hirschmann-Laborgeräte, Germany). Plasma was isolated after centrifugation (Haematospin, Hawksley, UK) for four minutes and plasma insulin was measured using a Ultra-sensitive Mouse Insulin ELISA (CrystalChem, USA) following manufacturer’s instructions. Area under the curve (AUC) was calculated by summation of trapezoids (Prism 8, GraphPad, USA). Using the matched plasma insulin and glucose values, HOMA-IR was calculated using the HOMA calculator (Diabetes Trial Unit, University of Oxford available here: https://www.dtu.ox.ac.uk/homacalculator/).

### Clinical lipid measures

Serum cholesterol, fatty acids, lipoproteins and gross TGs (Table S1) were measured as follows. Total cholesterol and total triglycerides were measured using a Dimension RxL analyzer (Siemens Healthcare Limited, UK). HDL cholesterol was measured using a homogeneous accelerator-selective detergent assay with a Dimension RxL analyzer (Siemens Healthcare Limited, UK). LDL cholesterol was calculated using the Friedwald equation [32]. Free fatty acids were measured using the Roche Free Fatty Acid Half-Micro Kit (Roche Diagnostics Limited, UK).

### Lipidomics

Lipidomics data for this study were drawn from a previous study that used a combination of mass spectrometry and phosphorus NMR to establish the lipidome of the six tissues used [33]. All procedures that were used to generate these data are therefore as described previously [33–35]. Briefly, whole tissue/organ samples were homogenised in a chaeotropic buffer to prepare a stable, pipettable solution that was then extracted with a mixture of dichloromethane, methanol and triethylammonium chloride, with adjustments for the abundance of triglyceride in adipose [33]. Samples of fresh diet were dispersed in the same chaeotropic buffer used for preparing tissue samples and homogenised thoroughly before being freeze-thawed and agitated (3× cycles, -80/40 °C). Chow diet samples were also centrifuged briefly (20k × *g*, 2 min) after homogenisation in order to collect debris-free supernatant. Homogenates were prepared by a different person to the dissections in order to blind the researcher to the groupings.

Mass spectrometry samples were prepared and data collected in a high throughput fashion using samples run in randomised order by Direct Infusion Mass Spectrometry [33, 35], via glass-coated 384 well plates. NMR samples were prepared and data collected in a low throughput fashion using a modified form of the CUBO solvent system and assigned using reference 2D spectra acquired for the purpose [33, 34]. Dual spectroscopy identified up to 776 lipid variables in positive ionisation mode and up to 467 lipid variables in negative ionisation mode in liver, brain, heart, vastus lateralis muscle and adipose tissue homogenates and in serum. The combination of the two spectroscopic techniques enabled us to verify the abundance of lipid classes that can be isobaric. PC and PE isoforms can be isobaric in positive ionisation mode, but are separable in both ^31^P NMR and negative ionisation mode, enabling identification without use of LC-MS.

### Lipid traffic analysis

Lipid Traffic Analysis code v1.0 [27] was further developed in the present study to produce Lipid Traffic Analysis code v2.3. The code for the Binary Traffic analysis (Switch Analysis) was updated to include alignment of lists and automated calculation of *J* and *p-*values from binary lists and improved categorisation of lipid variables (including assessment of all TG-derived glycerides). The configuration of the ***U***-lipid, ***A***-lipid and ***B***-lipid sections of the code was altered to make running any of the three individual parts of the code feasible. Novel code was written in R(v3.6.x) and processed in RStudio(v1.2.5x). See the *Data Availability Statement* for access to the full R code for Lipid Traffic Analysis v2.3.

In the analysis of the present study tissues used were mapped to the known biological/metabolic network (Fig. 1B) and lipids categorised in the Switch Analysis under ***A***, ***B*** and ***U*** types [27, 28]. Variables were regarded as present if they had a signal strength >0 in ≥66% of samples per group.

### Statistical methods

Univariate and bivariate statistical analyses, and error normalised fold change (ENFC) [27], were calculated in Microsoft Excel 2016. Graphs were prepared in Excel 2016 or OriginLab 2018. Calculations of Jaccard-Tanimoto Coefficients (JTCs, *J*) and associated *p-*values [27] were used as a non-parametric measure of the distinctions between lipid variables associated with phenotype(s). The *p*-value associated with each *J* represents the probability that the difference between the lists of variables for the two phenotypes occurred by random chance, representing both the number of variables only found in either of the two groups and the order of the binary list. All lipidomic data were assumed to be unequally distributed and heteroscedastic and so the appropriate type of non-parametric test was applied.

Glucose tolerance, body composition and liver weight data were normally distributed, and therefore unpaired Student *t*-tests were used.

## Results

### Mouse model of gestational diabetes

**Fig. 2:**
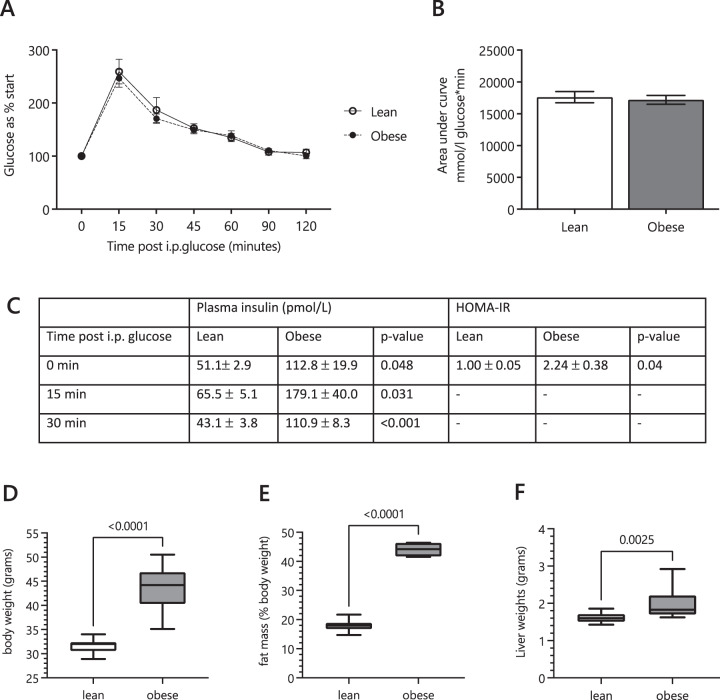
Glucose tolerance test and gross body composition of the mouse model at the time of tissue collection. **A** Blood glucose concentrations in post-weaning dams during a glucose tolerance test (GTT). **B** Glucose area under the curve during the GTT. **C** Insulin concentrations in dams’ plasma at basal (0), 15 and 30 min post glucose challenge and calculated fasting HOMA-IR (0 min). **D** Dam bodyweight at weaning. **E** Dam fat mass (measured by TDNMR) as a percentage of bodyweight at weaning. **F** Dam liver mass at weaning. The *p* values are based on unpaired Student *t*-tests between the lean and obese groups for comparisons given above braces between distributions. I.P., Intra-peritoneal; HOMA-IR, HOmeostatic Model Assessment for Insulin Resistance.

**Fig. 3:**
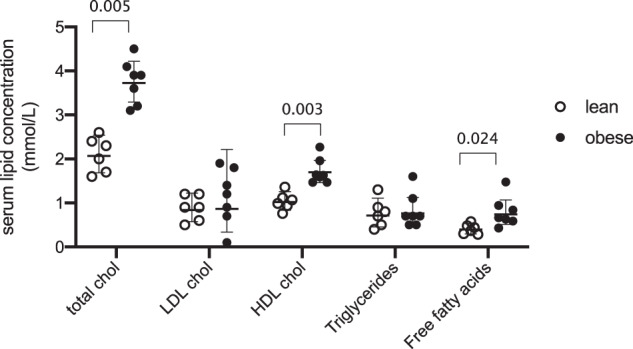
Lipoprotein, cholesterol and triglyceride measures. Determined using standard clinical bioassays. Values given in Table S1. The *p* values for comparisons given above braces between distributions. Chol, cholesterol.

Glucose tolerance tests showed that the lean (Control) and obese-GDM dams displayed similar glycaemic control *post partum* (Fig. 2A, B). However, serum insulin levels were increased in the obese group at all time points during the GTT (Fig. 2C), suggesting the obese-GDM mice were euglycaemic but insulin resistant. The obese group were, as expected, heavier and fatter that controls (Fig. 2D, E) and their livers were also heavier (Fig. 2F). The serum lipoprotein profiles of the two groups were distinct, with the obese-GDM group showing an increase in total cholesterol (mainly in HDLs) and increased circulating fatty acids (Fig. 3). This suggested that lipid metabolism differed between the two groups but does not explain how. We therefore investigated lipid metabolism at a molecular level. We began by testing the hypothesis that these effects were driven by a difference in the dietary intake of the two groups.

### Dietary fatty acid intake

Profiling of the fatty acid (FA) composition of the chow and high fat diets showed that the FA intake of the two groups was very different, however, included the same 33 FAs. Of the 33 FAs detected, 26 were highly significantly (*p* < 0.001) different between the two diets (Bonferroni correction for multiple variables *p* = 0.00152; Supplementary information 1). Lean mice ate around 5.7 g of chow per day (2.56 kcal/g; fat, 7.42% [kcal]; RM1, Special Diets Services, Witham, UK) whereas obese mice ate around 5.5 g of the HF pellet (6.79 kcal/g; fat, 45% [kcal]; 45% AFE Fat, Special Diets Services, Witham, UK) as well as 3 g of condensed milk per day (8% fat [*w/w*]; Nestle, fortified with mineral and vitamin mix AIN93G), with the latter consuming a higher overall fat intake than the chow-fed mice. We therefore tested whether any differences in abundance between the two groups might be attributed solely to dietary intake or whether there was a difference in control of FA metabolism and distribution in the lean and GDM systems. This was tested in storage (adipose) and oxidative (heart) tissues which represent a considerable fraction of all FAs within the organism but also represent tissues that are at a distance from dietary intake and thus their composition is subject to the controls of lipid metabolism in the organism. Thus, with adipose and heart, dietary FAs are so far removed that the diet will influence but not reflect or dictate the composition of the tissue. Our hypothesis was that the TG profile of the adipose tissue would be modulated to reflect the influx of the most abundant FAs of the dietary intake.

**Fig. 4:**
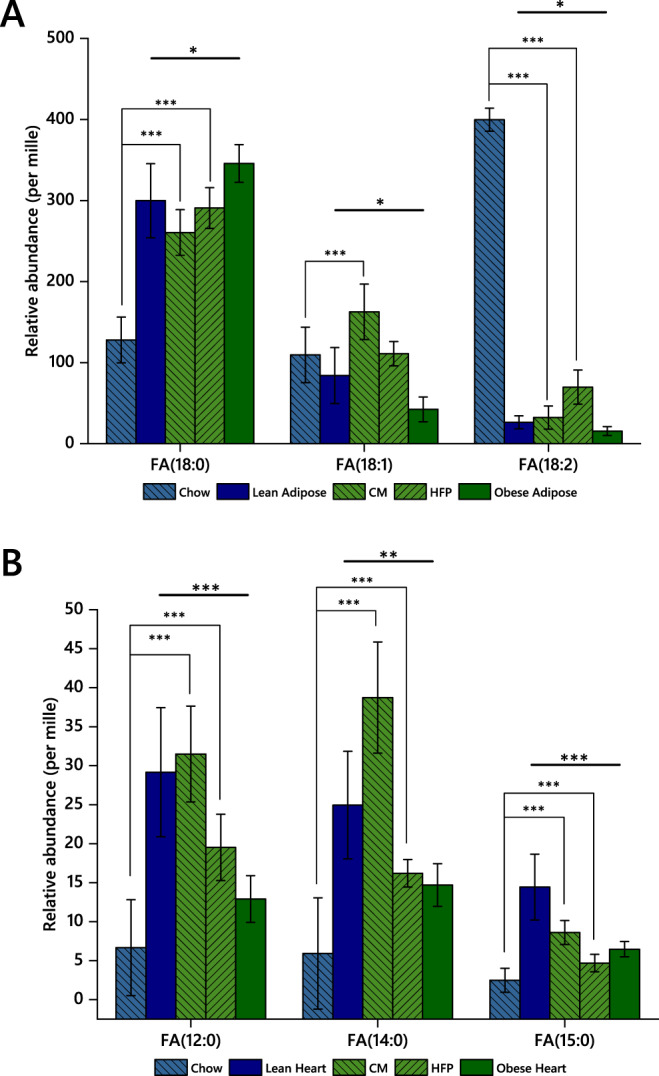
Relative abundance of FAs in chow and high fat diets and tissues from lean and obese groups. **A** Adipose tissue. **B** Heart tissue. *p < 0.05; **p < 0.005; ***p < 0.001. CM, condensed milk; FA, fatty acid; HFP, high fat pellet.

The relative abundance of three C_18_ FAs differed (*p* < 0.05) between the two groups in adipose tissue (Supplementary information 2). The abundance of FA(18:0) in adipose tissue from the lean group was just under three times that of chow diet, whereas the composition of FA(18:0) in the obesogenic diet was at least twice as high as that of lean dams (weight-for-weight, Fig. 4A). As the overall fat intake was higher for the HFD (obese-GDM) group, the accumulation of FA(18:0) in the adipose tissue of the obese-GDM group did not reflect this, being only ~10% higher. FA(18:1) was more abundant in the GDM group’s diet but the abundance in their adipose tissue was lower than that of lean dams (Fig. 4A). The abundance of FA(18:2) in the HFD was only about 10% of that of the chow diet, whereas the abundance of FA(18:2) was higher in the adipose tissue of obese dams. These results suggest that the way adipose tissue fatty acid distribution is controlled differs between the two groups and differences do not simply reflect differences in dietary composition. The composition of the adipose tissue represents the summative effect of both dietary intake and FA use. We therefore tested the same hypothesis in a tissue that typically use FAs as its major energy source and does not typically store them.

In heart tissue, which uses FAs as an energy source, two FAs showed very significant differences in mean abundance (*p* < 0.001) between lean and obese groups and one further, FA(14:0), with a *p* = 0.003, Fig. 4B. The differences here were more pronounced than those of the adipose tissue (*vide supra*, Fig. 4A). For all three of these FAs, the heart tissue of lean mice had higher relative amounts of FA(12:0, 14:0, 15:0) than that of obese-GDM animals, and the abundance of all three FAs was lower in obese hearts despite being more abundant in the obesogenic diet (Fig. 4B). Furthermore, the ratio of FA(15:0)/FA(17:0) was higher in the obese group (3.7 in obese and 2.4 in lean, *p* = 0.006), showing that the HFD increases the relative abundance of FA(15:0) relative to FA(17:0), consistent with a higher intake of dairy fat [36–38]. There was also a disparity in FA(16:0) (*p* = 0.003), the most abundant FA in the heart. FA(16:0) was around 10% more abundant in hearts from the obese group (Supplementary information 2).

These findings suggest that the control of FA accumulation and distribution of the four most abundant fatty acids, FA(16:0, 18:0, 18:1, 18:2), and at least three more minor ones, differed between the two groups. This strongly implies that the differences observed do not result solely from differences in the FA profile of dietary intake but are mediated by differences in control of lipid metabolism in the two groups of dams. Furthermore, the different effects observed in the heart and adipose compartments suggested that comparisons in the 2 tissue types would provide only a fragmentary pattern of lipid metabolism and that an integrative approach would be required to construct a more complete picture. In order to test the primary hypothesis that lipid metabolism differed systemically between the lean and obese-GDM mice, we progressed to a network analysis (Lipid Traffic Analysis) to characterise the lipid metabolism throughout the metabolic network of the two phenotypes by including an expanded set of tissues, *e.g.* liver, muscle and brain as well as serum.

### Lipid traffic analysis

**Fig. 5:**
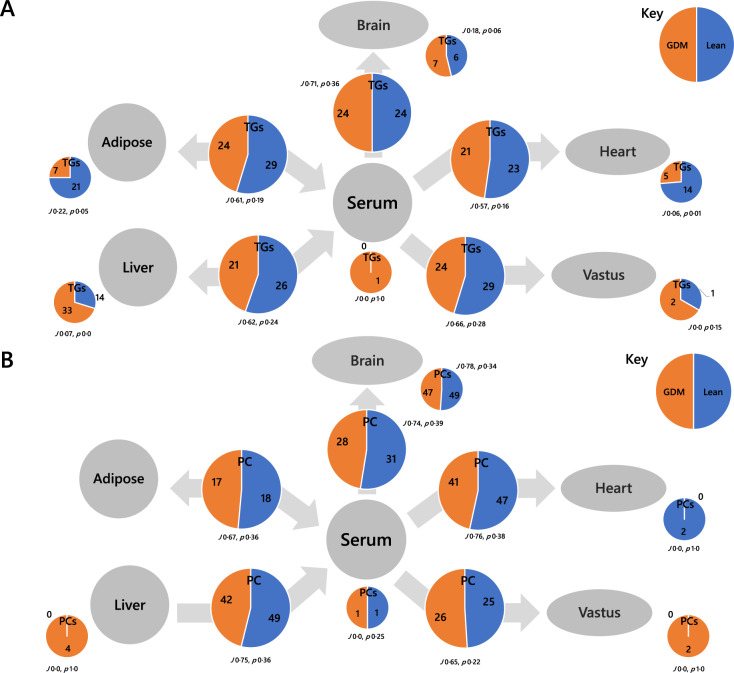
Switch analysis of the tissue network used in the present study. **A** Triglycerides (TGs), **B** Phosphatidylcholines (PCs). Small pie charts represent the numbers of variables only found in the given tissue, larger pie charts represent the numbers of variables found in both adjacent tissues. *J* represents the Jaccard-Tanimoto distance and *p* the accompanying probability that the binary list of variables for the two groups differed by random chance [27].

The Switch Analysis part of a Lipid Traffic Analysis [27, 28] (updated v2.3, see *Methods*) was used to plot the distribution of lipid variables throughout the biological network shown in Fig. 1B. The accumulation/absence of variables across the network indicates how the control of metabolism differs between the two groups. The Switch Analysis of TGs showed that there were generally more variables associated with the lean group, indicating a wider variety of TGs through the network in these animals (Fig. 5A). The TG variables between two adjacent compartments (***B***-type lipids) were associated with Jaccard-Tanimoto co-efficients (*J*) of around 0.65 with accompanying probabilities (*p*) of <0.4. Where *J* = 0.65, about two thirds of variables are the same for both phenotypes. Where *p* < 0.5 for this type of comparison, both groups being compared have variables that the other does not. Importantly, this is distinct from the use of *p* values in tests for quantifying the consistency between the null hypothesis and the tested hypothesis. The *J* ~0.65 *p* < 0.4 for ***B***-type TGs (Fig. 5A) show that the similarity between the two groups is not strong, consistent with the evidence of different distribution suggested above (Fig. 4). The same trend is observed in PCs, with *J* ~0.72 and *p* < 0.4 throughout ***B***-type PCs (Fig. 5B). This shows that the control of lipid metabolism is different in the two phenotypes, for both structural lipids (PLs) and energy supply and storage (TGs).

**Fig. 6:**
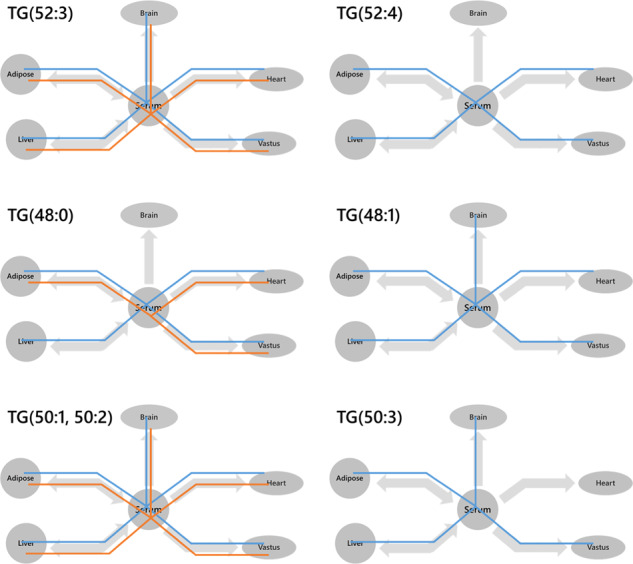
Wiring (London Underground) diagrams of triglyceride (TG) variables found in which tissues. Blue lines represent the lean group whereas orange lines represent the obese-GDM group. A variable was considered present if *B* = > 0.66.

The difference in variety and number of ***A***-type TGs and PCs led us to explore how lipid traffic differed between the groups. Isoforms of some of the most abundant TGs such as TG(52:3) appeared throughout the networks of both groups (Fig. 6). This was also true for TGs that are associated with *de novo* lipogenesis (DNL), such as TG(50:1) and TG(50:2) [39]. However several isoforms of TG were only found throughout the lean network and not the obese-GDM one, including TG(48:1, 50:3, 52:4). This indicated that a variety of TGs differed between the groups, suggesting that distribution as well as biosynthesis differed between the two phenotypes.

These results were echoed in the variables for structural lipid classes such as PCs, phosphatidylethanolamines (PEs) and sphingomyelins (SMs). Although several commonplace palmitate (FA(16:0)) and oleate- (FA(18:1)) containing isoforms of PC appeared throughout the networks of both phenotypes (Fig. S1), the adducts detected suggest that some are less abundant in the obese group, e.g. chloride adducts of PC(36:3, 38:5). Despite this, some polyunsaturated PCs are found widely across the network, such as the DHA-containing PC(36:6, 40:6), suggesting subtle but important changes in the control of FA composition of PCs. The contrasts were clearer in PEs, where although PE(36:1, 36:2, 36:4) are found throughout, PE(34:2) was found throughout obese systems whereas PE(34:0) was in lean mice (Fig. S2). This suggests that the gross PE composition of the systems differed between phenotypes, moving to PE(34:2) and from PE(34:0) in the obese-GDM group.

Several isoforms of SM, 33:1, 34:1, 36:1, 37:1, appear throughout most or all of the network for both phenotypes, but at least two isoforms appear throughout only one. For example, SM(39:1) appeared throughout the obese network but not in the lean, and SM(35:1) appeared throughout the lean phenotype but not in the obese (Fig. S3). SM(39:1) is a known marker of dairy intake, consistent with the composition of the obesogenic diet, however what directs the appearance of SM(35:1) only in the lean phenotype is unclear. Despite structural similarities to SM(39:1) and SM(35:1), SM(37:1) appears almost throughout both networks, suggesting no connection with diet or phenotype.

The lipids that were unique to a particular compartment, i.e. ***U***-type lipid variables, also provided evidence that supported the hypothesis that disrupted lipid metabolism follows a pregnancy characterised by GDM. We found that there were several ***U***-type TGs in adipose tissue, with about three times as many in adipose tissue of the lean group (21:7, *J* 0.22, *p* 0.05; Fig. 5A). This was similar for hearts (14:5, *J* 0.06, *p* 0.01; Fig. 5A). However, the situation was inverted for liver, where there were more ***U***-type variables for the obese than the lean group (14:33, *J* 0.07, *p* 0.00; Fig. 5A). This led us to compare the TG isoforms present in the obese liver and lean heart/adipose in order to determine whether there was a connection. Six of the variables involved were found in both the livers of the obese group *and* the hearts of the lean group, TG(40:00, 40:01, 42:00, 46:02, 50:05, 52:05). This group of variables comprised isoforms associated with *de novo* lipogenesis, TG(40:00, 40:01, 42:00, 46:02), and polyunsaturated species more closely associated with dietary intake, TG(50:05, 52:05). Thus, the spatial distribution of a range TGs differs between the two phenotypes, with a possible restriction on TGs being transported out of the liver in the obese phenotype.

Although this accounts for 6 variables, there are around 20 more ***U-***type TGs in obese than lean livers. A further comparison of the isoform lists suggests that these are mainly odd-chain containing TGs with little overlap with lean heart or adipose (Table S2). The presence of such a variety of odd-chain-containing TGs suggests that several such TGs are produced locally. There are also a variety of odd-chain-containing PCs on the serum-brain axis that appear in both phenotypes (Fig. 5B), suggesting that odd-chain fatty acid metabolism is not necessarily a hallmark difference of these two phenotypes.

## Discussion

The aim of this study was to test the hypothesis that changes in control of lipid metabolism outlast the hyperglycaemia associated with GDM. In the mouse model used, glycaemic control during a glucose challenge after weaning did not differ between lean and obese-GDM dams at the end of lactation, but the post-GDM group remained insulin resistant. Comparison of FA intake with the FA composition of individual tissues showed that the FA composition in tissues differed considerably between groups and that differences in dietary intake were not the primary driver of this effect. Lipid Traffic Analysis showed that the lean group had a wider variety of both TGs and PCs than the obese phenotype, and that there was different lipid traffic through the lean and obese animals, with some lipid variables being retained in the liver in the GDM phenotype but being found in the heart in the lean phenotype. Taken together, these results showed that altered control of lipid metabolism and insulin resistance were features of post-GDM obese dams. This combination of metabolic dysregulations is of interest because the obese-GDM phenotype represents a group at much higher risk of metabolic disease, such as T2DM, after pregnancy. The cause of this increased risk is presently unknown. Humans display evidence of dysregulation of lipid metabolism and distribution in pregnancy before development of insulin resistance [10–12]. Therefore our working hypothesis is that dysregulated lipid metabolism drives the insulin resistance/hyperinsulinaemia that remains *post partum* and contributes to increased future risk of T2DM after GDM.

A relationship between lipid metabolism and insulin resistance is well established. Insulin resistance such as that found in the present study is accompanied by raised circulating FAs (as a consequence of adipose tissue insulin resistance), which inhibit glucose uptake into skeletal muscle [40–42]. Furthermore, lipid ‘spill-over’ from adipocytes is more frequent in obesity, with adipocytes exhibiting higher rates of spontaneous lipolysis [43], resulting in increased delivery of fatty acids and triglyceride production in non-adipose tissues. This is reflected in the current study by an increase in liver weights of the obese dams and the appearance of ‘obese-specific’ liver TGs associated with *de novo* lipogenesis. A strength of the present study for understanding the relationship between insulin resistance and lipid metabolism is that it analyses the difference in lipid metabolism between a healthy (lean) post-pregnancy group and one that developed GDM.

This study found evidence that both TGs and PCs have different isoform profiles and a different number and variety of variables throughout the lean and obese animals. Such a difference between groups raises questions about how the system operates. Indeed, there are several possible explanations for such different lipid profiles. The supply of FAs is an obvious possible driver of FA abundance in vivo. However, although the abundance of FAs in the diet of the two groups was distinct, the accumulation and distribution of FAs could not be explained by the composition of the diet. Paradoxically, the lean group displayed a greater variety of lipids throughout their systems despite all of the same FAs being detected in the chow and high fat diet, and fat intake being considerably higher in the HFD group. Broadly, there are two possible explanations for a different breadth of variety of lipids in the presence of an adequate supply of FAs and similar physical activity. One is that endogenous production of FAs (e.g. *de novo* lipogenesis) is more nuanced where there is a greater intake of carbohydrate (as there is in the chow diet), and thus this improves the availability of FAs. The other is that the control of lipid biosynthesis from the formation of diglyceride differs between the two groups. Either way, how FAs (either alone or in DGs) are marshalled differs between the two groups, altering the range of lipid isoforms observed.

FA variety may arise partly from fatty acid modification. Several modifications of FAs are possible in vivo, including desaturation, chain lengthening (elongation) and chain shortening. The latter typically produces fatty acids with an odd number of carbons. The possibility of differences in FA modifications between groups is intriguing as there is long-standing evidence for a relationship between fatty acid metabolism, in particular the better distribution of the long-chain polyunsaturated arachidonic acid and its hydroxylated derivatives, and the development of insulin resistance [44, 45] (reviews [46, 47]). Greater endogenous synthesis or a better distribution of arachidonic-acid containing lipids (such as PC [48]), as observed in the lean group, may therefore contribute to a return to normal insulin sensitivity in that group. Indeed, the evidence for a difference in distribution of arachidonate-containing isoforms of PC is particularly important where the supply of arachidonate is not restricted, as in the present study. The supply of arachidonic acid is not expected to be a metabolic bottleneck where there is a good dietary supply of it or linoleic acid (as in the current study), as linoleic acid can be lengthened and desaturated to produce arachidonic acid in mice and humans [49, 50]. However, how it is marshalled through the system may differ, e.g. PC(38:5), Fig S1.

Odd-chain fatty acids (OCFAs) have been associated with lower risk of T2DM [51, 52] (review [53]). This is typically associated with a higher dietary intake of dairy fat [54], however it is difficult to test hypotheses based on this directly as it includes a number of species, principally FA(15:0) and FA(17:0), that can come from both dietary and endogenous synthesis, e.g. FA(17:0) from the product of *Hacl1*. FA(15:0) can come from dairy fat [36], for example through branched FAs produced by Gram-positive bacteria in the gut of *Bos taurus*. The presence of these and other OCFAs could increase the number of lipid isoforms detected. Importantly, OCFA-containing lipids appeared throughout the system.

A number of OCFA-containing PCs were found in the brains of both groups, suggesting that OCFAs are part of normal lipid metabolism in this tissue, and unaffected even by considerable differences in dietary intake. OCFAs are also commonplace in TGs. In the present study OCFAs appeared in a variety of tissues in both feeding groups but with little overlap between compartments, indicating that circulation of OCFA-containing species is poor or that they are generally made locally. However, labelling studies would be required to establish this formally. The mechanistic connection between any single or all OCFAs, and the risk of insulin resistance is unknown. Investigations based on circulating lipid composition showed that there is evidence for separate derivation of FA(15:0) and FA(17:0) [36–38] consistent with both endogenous and dietary influences. However, the effect of FA(17:0), the one produced in greater amounts endogenously, is stronger [55]. The biochemical role of these fatty acids has yet to be elucidated, including whether they represent cause or effect.

The difference in lipid variety between the two animal groups can also be explained by a difference in the control of lipid metabolism. One possibility is that although the obese-GDM group had excess fat intake, including the saturated FAs (SFAs) associated with DNL, their livers did not reduce or discontinue production SFAs from excess carbohydrate, leading to increased abundance of FA(16:0)- (palmitic acid) containing lipids, with knock-on effects on the relative abundance PUFA-containing lipids. This contrasts with the lean animals for whom endogenous biosynthesis of palmitic acid is important because their diet is naturally low in it. A strength of the present study is that the system-level analysis enabled us to separate local and system-wide differences between groups and thus identify the reach of lipids associated with particular processes, such as DNL, or with clear roles such as structural components of membranes. This was useful for both TGs and PLs.

The range of TGs associated with *de novo* lipogenesis (DNL), TG(48:0, 48:1, 50:1, 50:2, 50:3) [39], was narrower in the obese-GDM group, with TG(48:1, 50:3) not found throughout the latter group (Fig. 6). This difference in variety existed despite the total TG abundance in the circulation being the same for both groups (Fig. 3), suggesting that how the control of the mixture of TGs, and thus FAs differed between the groups. A clear difference in system-wide traffic of structural lipids was also observed clearly. For example, saturated PE(34:0) was found throughout most of the lean system whereas it was not in the obese-GDM group, whereas the unsaturated PE(34:2) was only found throughout the obese system.

The kind of systemic analysis used on the mouse model in the present study is probably impossible in humans, however a comparison of the systemic changes in lipid metabolism that we observed in the current study and those observed in the circulation of obese humans before GDM was diagnosed [10–12] showed that there were several similarities between the two. Polyunsaturated PCs were less abundant in the circulation of obese humans before GDM diagnosis [11] and in the obese dams *post partum* (Fig. S1). Furthermore TGs associated with DNL were reorganised in both humans before GDM [11, 12] and in the current study two known markers of DNL, TG(50:1, 50:2) were maintained throughout the systems of the obese-GDM group, and were also higher in obese humans who later developed GDM [11, 12]. These similar shifts before diagnosis of GDM diagnosis (humans) and after delivery (mice) led us to the hypothesis that altered systemic lipid metabolism contributes mechanistically to the development of insulin resistance. Results from an observational study of the progress of GDM to T2DM in humans agrees with these direct observations, also finding the same molecular markers associated with GDM after pregnancy and for progress to T2DM [56], as the earlier studies found in advance of hyperglycaemia in human pregnancy. This would explain the glucose intolerance in situations of increased insulin resistance, such as that associated with pregnancy (GDM) or with ageing (T2DM). Therefore, dysregulation of lipid metabolism could precede and contribute to the development of dysregulated glycaemia and ultimately glucose intolerance.

**Fig. 7:**
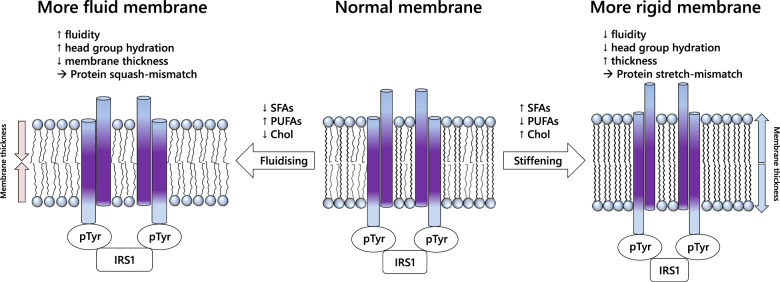
Summary of the hypothesis that the increased risk of CVD/T2DM associated with obese GDM is associated with altered lipid metabolism.

The difference in the composition of structural lipids in membranes provides a possible mechanism for these effects. For example, the differences in PC and PE composition and supply discussed above suggest that there are changes in both bilayer (PC, SM) and non-bilayer (PE) lipids, with important implications for regulation of membrane fluidity [48, 57]. The relationship between membrane composition and membrane protein activity is well established [58, 59]. Specifically, physical studies of membrane behaviour show that where there is an increase in the abundance of saturated and mono-unsaturated FAs of medium length (16 or 18 carbons) and thus a proportional reduction in the abundance of shorter-chain and polyunsaturated FAs, the membrane will become less fluid [60, 61], an effect known to modulate the kinetics of enzymes and the affinity of receptors for substrates [62, 63]. Theoretically, both a more and a less fluid membrane could therefore disrupt the activity of insulin receptors (for example), with physical effects caused by membranes that are either too fluid or too rigid. This is shown schematically in Fig. 7. This hypothesis provides an explanation for insulin resistance being driven by a dysregulation of lipid metabolism, and for observations that rigid membranes impair insulin signalling [64]. This is consistent with the hypothesis that emergence of GDM and T2DM is not driven solely by short-term over-nutrition.

In conclusion, the present study showed that obesity-induced GDM driven by a high fat-high sugar obesogenic diet is associated with considerable changes in the systemic control of the lipid distribution. This included tissue-specific effects on lipid metabolism that were not attributable to differences in dietary intake but could affect insulin sensitivity. The present study therefore shows that unlike the hyperglycaemia present during GDM [15], changes in lipid metabolism associated with GDM persist post*-*weaning. Therefore metabolic dysregulation of lipids provides a mechanism for the development of T2DM in women previously affected by GDM. We propose that dysregulation of lipid metabolism, associated with obesity and GDM, contributes to the observed increased risk of T2DM and cardiovascular disease in women who have had one or more GDM pregnancies [65]. As cardiovascular disease is the number one killer of women in the UK and the US, with almost double the mortality rate of breast cancer [22], we conclude that studies such as this are key to identifying potential targets for intervention to prevent the increasing burden of poor cardio-metabolic health.

## Supplementary information

List of supplementary files

Supplementary figures and tables

Supplementary information file 1. FAs in feeds

Supplementary information file 2. FAs in feeds, heart and adipose

Supplementary information file 3. LTA v2.3 R Code

## Data Availability

The novel R code developed in the present study for Lipid Traffic Analysis v2.3 is in Supplementary Information S3. LTA v1.0 is publicly available [27, 66]. The MS dataset used in the present study is available publicly, as are the original NMR data [33].
