# Supplementary material for: A mouse model of gestational diabetes shows dysregulated lipid metabolism post-weaning, after return to euglycaemia
Source: Nutr Diabetes. 2022 Feb 15;12:8. doi: 10.1038/s41387-022-00185-4 (PMC8847647; doi:10.1038/s41387-022-00185-4)
Supplement: Supplementary file 1 — List of supplementary files [file 41387_2022_185_MOESM1_ESM.docx]

**Supplementary Files for Furse & Fernandez-Twinn *et al.* 2022.**

1. Supplementary Figures (.docx
2. Supplementary information file S1. FA composition of diets and statistics
3. Supplementary information file S2. FA composition of heart and adipose tissues and statistics
4. Supplementary information file S3. Lipid traffic Analysis v2.3 (R code for conducting Lipid Traffic analysis.)
