# Supplementary material for: A mouse model of gestational diabetes shows dysregulated lipid metabolism post-weaning, after return to euglycaemia
Source: Nutr Diabetes. 2022 Feb 15;12:8. doi: 10.1038/s41387-022-00185-4 (PMC8847647; doi:10.1038/s41387-022-00185-4)
Supplement: Supplementary file 2 — Supplementary figures and tables [file 41387_2022_185_MOESM2_ESM.docx]

**Supplementary Figures and Tables**

|  | Lean | | Obese | | *p* value |
| --- | --- | --- | --- | --- | --- |
|  | mean | s.d. | mean | s.d. |  |
| Total chol | 2.1 | 0.4 | 3.8 | 0.5 | 0.005 |
| LDL chol | 0.9 | 0.3 | 1.1 | 0.6 | 0.604 |
| HDL chol | 1.0 | 0.2 | 1.7 | 0.3 | 0.003 |
| TGs | 0.8 | 0.3 | 0.8 | 0.4 | 0.916 |
| FAs | 0.4 | 0.1 | 0.8 | 0.3 | 0.024 |

**Table S1. Clinical lipid measures.** Values plotted in *Fig. 3*. All measures in mmol/L.

| LIV  (obese) | ADI  (lean) | HEA  (lean) |
| --- | --- | --- |
| DG(23:00) |  |  |
| DG(27:00) |  |  |
| DG(27:01) |  |  |
| DG(31:02) |  |  |
| DG(33:02) |  |  |
|  | DG(35:01) |  |
|  | DG(35:02) |  |
|  | DG(35:05) |  |
| TG(33:00) |  |  |
| TG(35:00) |  |  |
| TG(37:00) |  |  |
| TG(37:01) | TG(37:01) |  |
| TG(39:00) |  |  |
|  | TG(39:01) |  |
|  |  | TG(45:01) |
| TG(47:01) |  |  |
|  |  | TG(49:00) |
| TG(49:01) |  |  |
|  | TG(49:02) |  |
| TG(51:02) |  |  |
| TG(51:03) |  |  |
|  | TG(51:04) |  |
|  |  | TG(51:05) |
| TG(53:03) |  |  |
|  |  | TG(55:01) |

**Table S2. Odd-chain containing triglyceride and triglyceride fragments.**

**
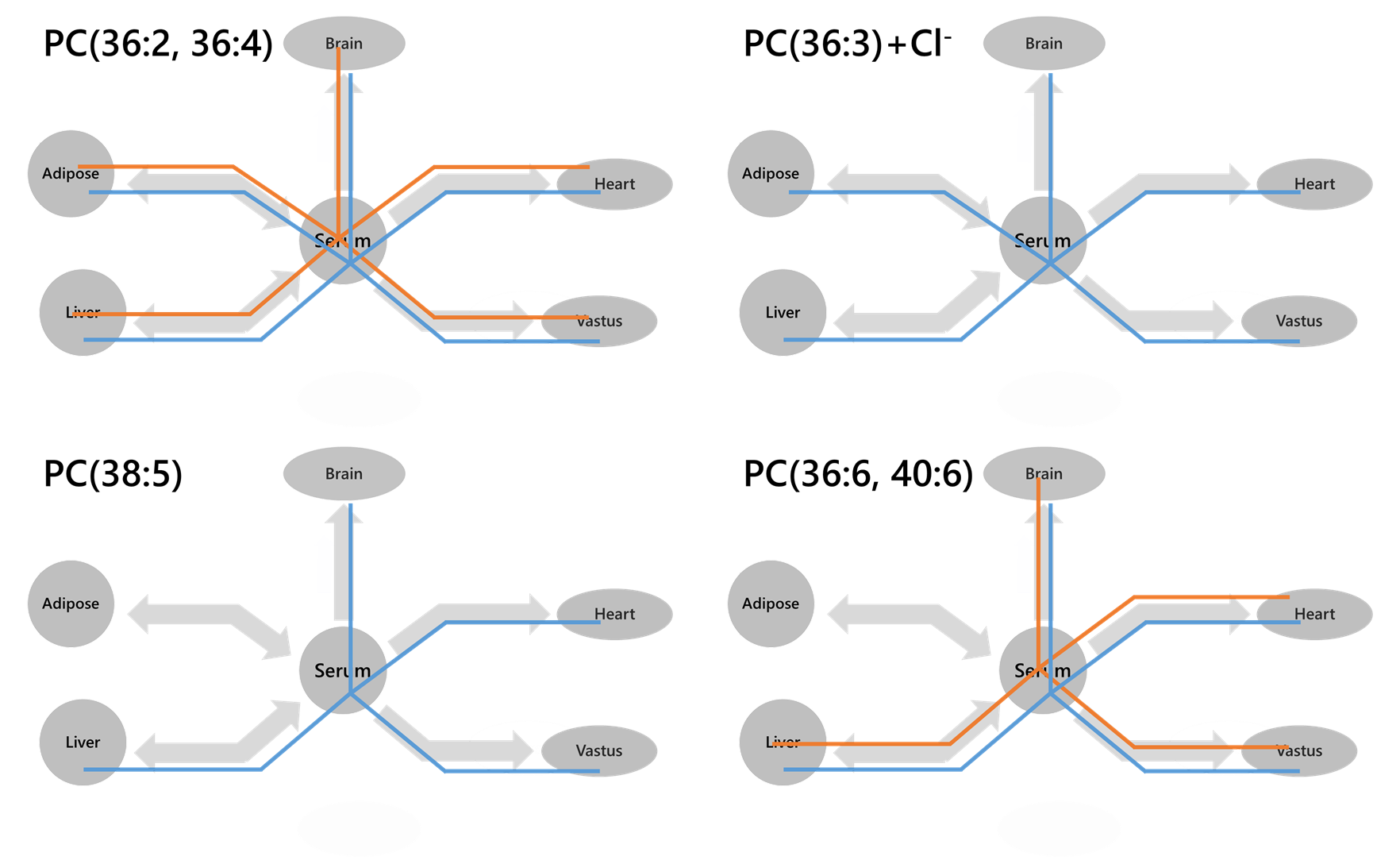
**

**Fig. S1. Wiring diagrams of phosphatidylcholine (PC) variables found in which tissues.** Blue lines represent the lean group whereas orange lines represent the obese-GDM group. A variable was considered present if *B* = >0.66.

**
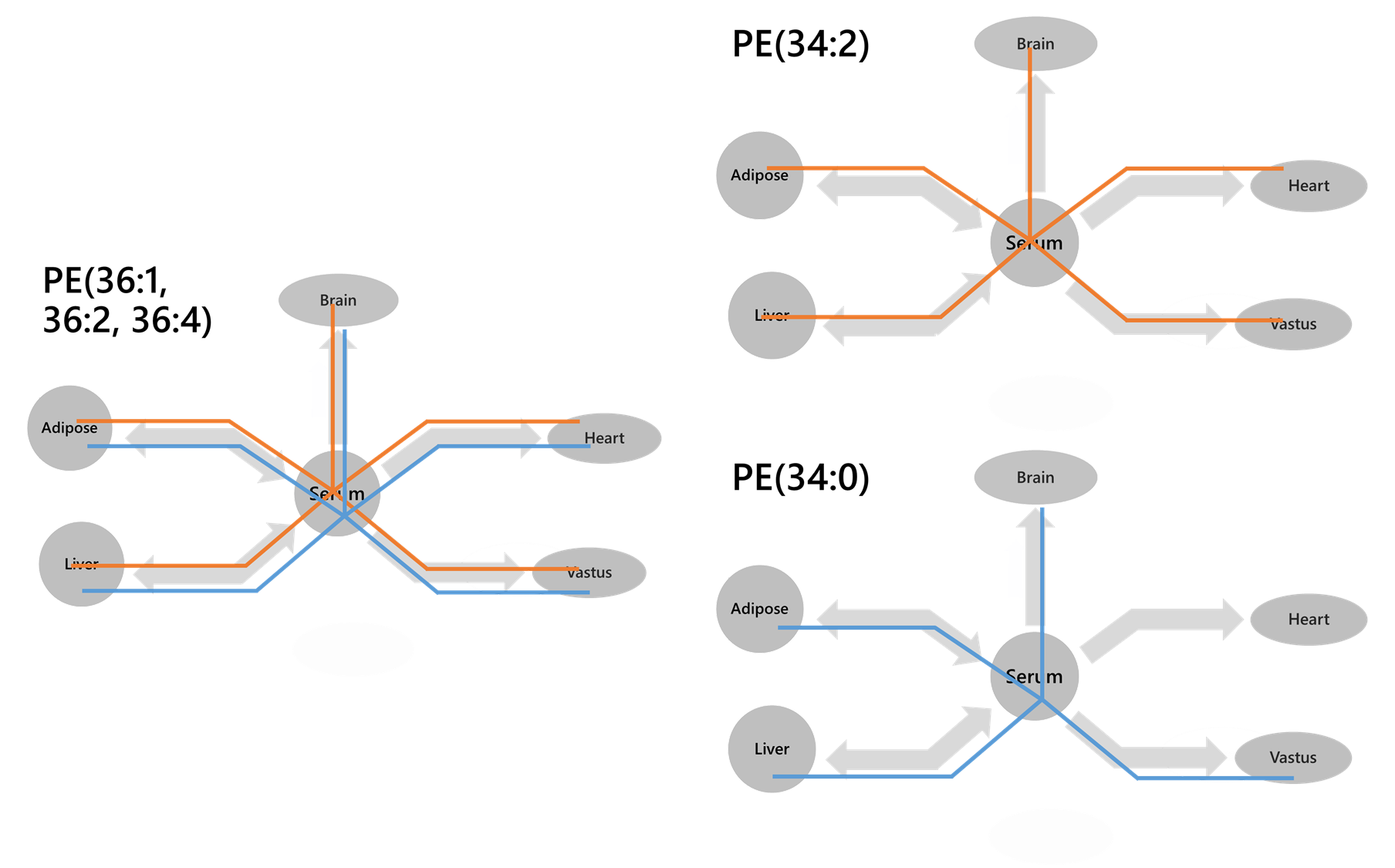
**

**Fig. S2. Wiring diagrams of phosphatidylethanolamine (PE) variables found in which tissues.** Blue lines represent the lean group whereas orange lines represent the obese-GDM group. A variable was considered present if *B* = >0.66.

*
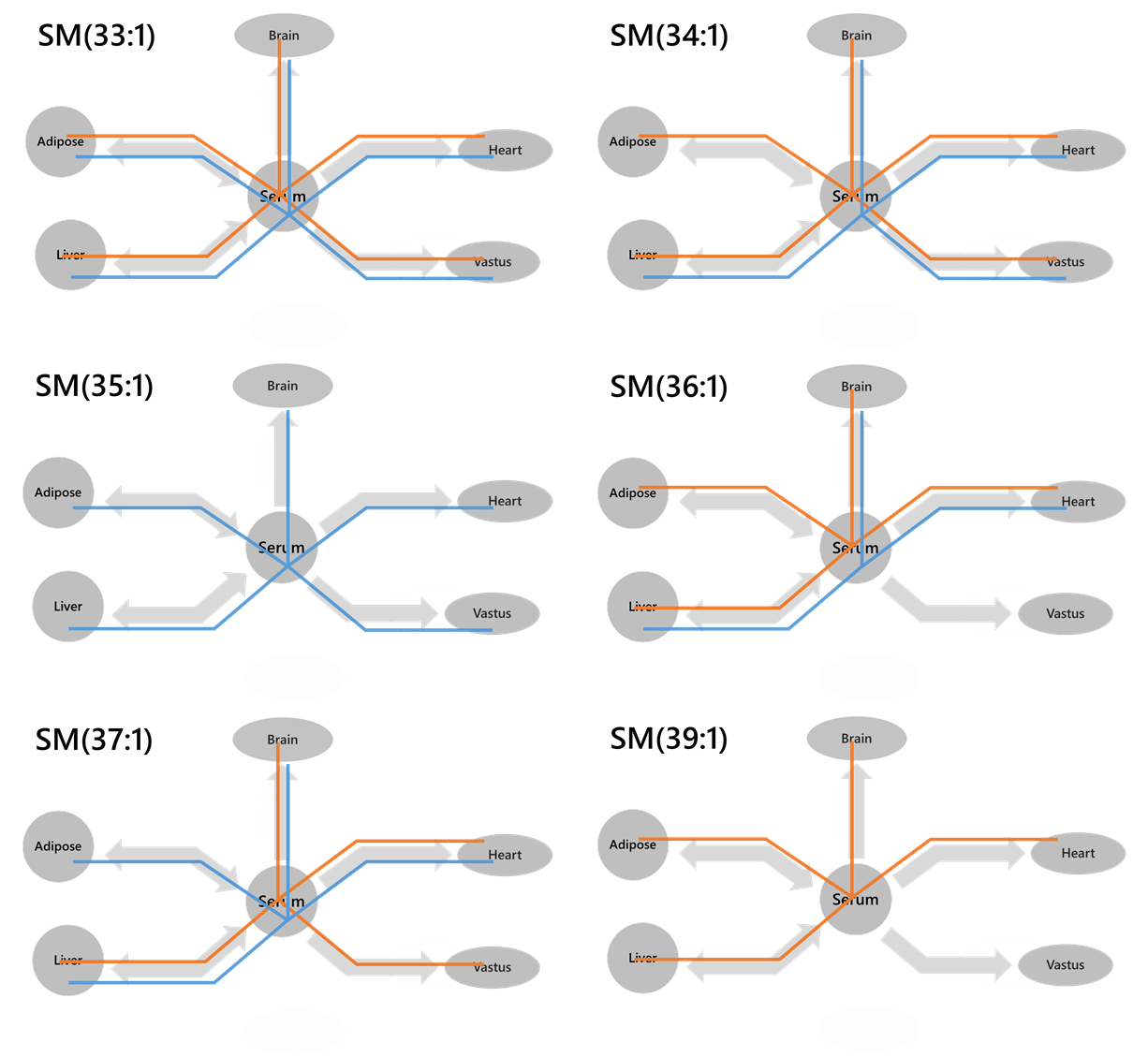
*

**Fig. S3. Wiring diagrams of sphingomyelin (SM) variables found in which tissues.** Blue lines represent the lean group whereas orange lines represent the obese-GDM group. A variable was considered present if *B* = >0.66.
